# Tibetan Medicine: A Systematic Review of the Clinical Research Available in the West

**DOI:** 10.1155/2013/213407

**Published:** 2013-04-11

**Authors:** K. Philip Reuter, Thorolf E. R. Weißhuhn, Claudia M. Witt

**Affiliations:** Institute for Social Medicine, Epidemiology and Health Economics, Charité-Universitätsmedizin, 10098 Berlin, Germany

## Abstract

*Background*. Little is known about Tibetan medicine (TM), in Western industrialized countries. *Objectives*. To provide a systematic review of the clinical studies on TM available in the West. *Data Sources*. Seven literature databases, published literature lists, citation tracking, and contacts to experts and institutions. *Study Eligibility Criteria*. Studies in English, German, French, or Spanish presenting clinical trial results. *Participants*. All patients of the included studies. *Interventions*. Tibetan medicine treatment. *Study Appraisal and Synthesis Methods*. Included studies were described quantitatively; their quality was assessed with the DIMDI HTA checklist; for RCTs the Jadad score was used. *Results*. 40 studies from 39 publications were included. They were very heterogeneous regarding study type and size, treated conditions, treatments, measured outcomes, and quality. *Limitations*. No Russian, Tibetan, or Chinese publications were included. Possible publication bias. *Conclusions*. The number of clinical trials on TM available in the West is small; methods and results are heterogeneous. *Implications of Key Findings*. Higher quality larger trials are needed, as is a general overview of traditional usage to inform future clinical trials. *Systematic Review Registration Number*. None.

## 1. Background

Traditional Tibetan medicine (TM), sometimes called “Lamaist” or “Buddhist” medicine, has developed in 1200 years into a unique medical system [1–3]. In TM, disease is understood as an imbalance of the three “Nyes-pa” (principles) consisting of one or two elements: “rLung” (air, wind), “mKhris-pa” (fire), and “Bad kann” (earth and water) [4]. Buddhist philosophy as well as shamanic origins of Tibetan culture form a background of cosmological, mind-body, and spiritual dimensions [1–3]. Treatment may consist of medicines (usually preparations of plants [5], seldom minerals or animals), physical treatments (e.g., massage, baths), life and diet regulation, or spiritual techniques [4]. Standardization of the originally individualized medicines, separation from the underlying philosophies, and discontinuation of some techniques (e.g., Tibetan dental medicine, cauterization) have led to derivative forms of TM [6]. We will use the term “Tibetan medicine” for the traditional TM (with its individual life style advice, diet, physical, and spiritual means) as well as larger or smaller subsets or varieties of it, down to single formulas.

**Figure 1:**
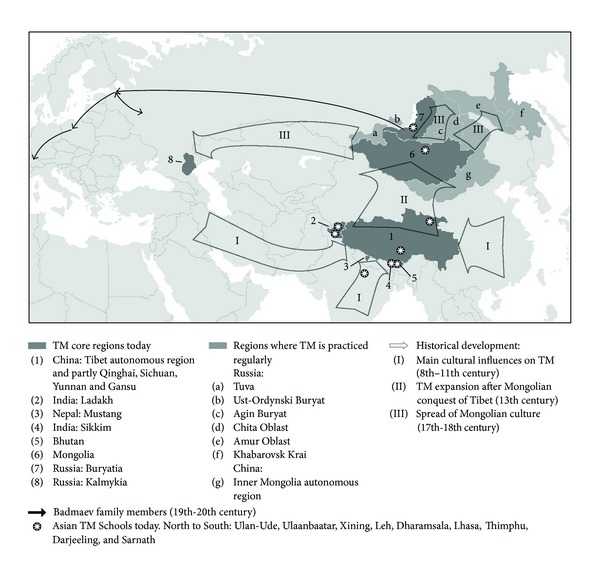
Tibetan medicine in geography and history. Map based on [7, 8, 17–22].

Besides the regions of the historical Tibet, very similar medical traditions are practised since the Mongolian conquest of Tibet in the 13th century in Mongolia, adjacent Siberia, and in the Russian province Kalmykia (Figure 1) [7]. Especially with traditional Mongolian medicine, TM has a substantial similarity. TM use in Western industrialized countries (the “West”) originates in a line of descendants of a Buryat physician migrating westward in the 19th century (Figure 1) [8, 9]. Still, there is little awareness of TM in the general Western public. Following the rising interest in traditional Chinese medicine (TCM) and complementary or alternative medicine (CAM) in general, more demand from Western countries can be expected in the future. The amount of available research in the West is small. A Medline search up to December 31, 2010, for example, for “Tibetan medicine” returned 371 hits, 0.0183 times the number for “traditional Chinese medicine.” The existing literature indicates a palliative, possibly curative potential, especially for chronic diseases [10], but studies on its multimodal individualized approach are scarce and systematic reviews exist only for one TM product [11–15]. Therefore, we attempted to present in this paper a systematic overview of clinical research currently available in the West on Tibetan medicine, and aim to provide details on methods and study quality. Some preliminary data can be found in [16].

## 2. Methods

A preliminary list of 15 literature databases was tested using the search terms “Tibetan medicine,” “Himalaya medicine,” “Tibetan herbal,” and “Lamaistic medicine.” The database list had been compiled from recommendations by experts, by Ovid [23], and by Deutsches Institut für Medizinische Dokumentation und Information (DIMDI) [24]. Those returning the most hits were used for the literature search, together with databases that were recommended by experts or appeared relevant in their characterization on the websites of DIMDI or the Charité library [25]. We finally searched seven databases up to publication date December 31, 2010: ABIM (accessed via Rijksuniversiteit Groningen), AMED (DIMDI), CAMbase (cambase), CCmed (DIMDI), Cochrane Collaborative Library (OVID), Embase (OVID), and Medline (PubMed). The search term “(Tibet OR Himalaya OR Mongolia OR Buddhist) AND (herbal OR medicine) AND study” was adapted as necessary to database language and syntax. Similar searches were used on the medical information services of DIMDI [24] and ZB MED [26] and by adding “AND clinical study” on Google scholar [27]. The published literature lists [28, 29] were screened. We also contacted European experts, research departments of TM medical faculties (Mentsekhang) in Lhasa and Dharamsala, and European centres for TM [30–32]. All identified literature was further screened for relevant citations. Duplicate references were eliminated throughout the process; of multiple publications of a study the most recent one was included. Included papers had to be written in English, German, French, or Spanish and had to present clinical trial results on a clinical outcome. No further restrictions were applied.

One of the authors (K. P. Reuter) used a predefined form to extract descriptive study data into MS Access 2003 and MS Excel 2003 [33, 34] data bases, including bibliographic data, and study parameters such as type, methods (including diagnostics, randomization, and blinding), and patient numbers. Furthermore, data regarding treated diseases, interventions, outcomes, and types of outcome measures (clinical symptoms, tests, and laboratory parameters) were extracted. If no primary outcome was defined, the first outcome mentioned in the title or the abstract was extracted, unclear cases were discussed with another author (C. M. Witt) until consensus was reached.

Methodological quality of the studies was determined with a DIMDI checklist (Table 1) that is used to evaluate studies for in-/exclusion in health technology assessments (HTA) in Germany [35]. The checklist has up to 31 items sorted into 7 categories and was used on a descriptive basis. Randomized controlled trials (RCTs) were further evaluated with the Jadad score [36, 37]. Descriptive statistics were calculated using MS Access 2003 and MS Excel 2003 [33, 34].

## 3. Results

**Figure 2:**
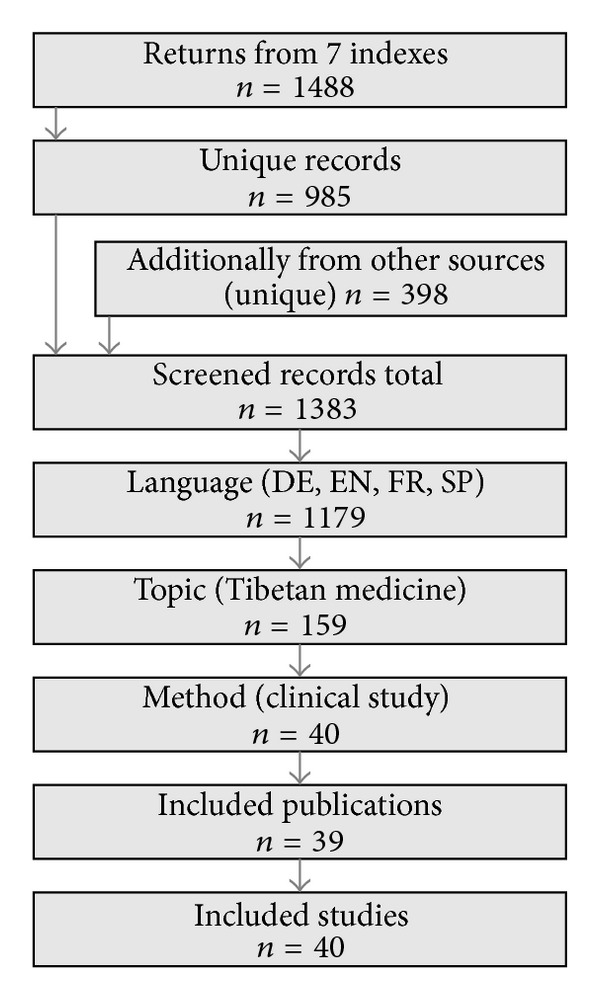
The literature search. References from indexing services were collected first, then other sources were added.

From 1383 screened records, we identified 40 studies reported in 39 publications (one contains 2 studies [38]), see Figure 2. An additional search without the terms “herbal,” “Buddhist,” and “Mongolian” did not result in fewer relevant publications. Thirty-five of the publications were journal articles, two were book chapters, and one is treated in this paper as a single Internet publication, although different findings had been published in several online media reports [39]. Only 18 publications were found by the initial data base searches. Most of the others were indeed indexed, as a reverse search (for already known publications) revealed. Written in English were 53.8% (*n* = 21) of the publications, the other 46.2% (18) were in German. Most publications came from Poland and Switzerland (30.8% or *n* = 12 each, all on products of Padma AG). The Asian studies were from India (15.4%, *n* = 6) or China (5.1%, *n* = 2). The earliest publication appeared in 1970. Since 1990 every 5 years about 3 new RCTs were published and, less evenly distributed, most of the observational studies (total *n* = 14). The 5 nonrandomized controlled trials were published between 1986 and 1991, and the 6 case studies or case series in 1998 or later (Table 2). The setting of 7 studies (17.5%) was multicentred [40–46]. Four studies (10.0%) were retrospective [40, 45, 47, 48].

In the RCTs included were 2028 patients, 1020 of them received the Tibetan medicine treatment. Study duration ranged from 14 days to 12 months (mean = 114 days). Most RCTs investigated Padma 28 (*n* = 9) (the first study in [38], and [49–56]) or Padma Lax (*n* = 1) [57]. A whole medical system approach with a complex traditional TM intervention was applied in 3 studies on diabetes mellitus [43], arthritis, [58] or hepatitis B [59]. Tibetan yoga in lymphoma patients [60] and a single TM preparation (Zhi Byed 11) for postpartum haemorrhage [44] were each the subject of 1 RCT. One study [61] was declared an RCT but lacked randomization.

From those publications including herbal medicines, four did not provide details on the used medication [42, 58, 59, 62], two provided the name of the preparations but not the ingredients [43, 48], and two provided the name of the preparation and ingredients, but no information on the quantity of the ingredients [44, 63]. Data on both ingredients and their quantity was only available for Padma 28 and Padma Lax. 

The duration of the non-randomized controlled trials was between 6 weeks and 6 months (mean = 43 d), 54% of the 678 patients received the verum Padma 28. Four non-randomized controlled trials included children with chronic respiratory tract infections [46, 64, 65] or juvenile arthritis [66]. One trial on adults included angina pectoris patients [61]. 

In the observational studies included, there were 1824 patients. The observation duration ranged from 15 days to 2 years (mean = 217 days). In some of the publications, the study duration was not clearly stated (the second study in [38], and [41]) or varied between participants [42, 45, 63]. Seven observational studies investigated Padma 28 (the second study in [38], and [47, 67–71]). One study each investigated Padma Lax [41] or a jewel pill (Byu-Dmar 13) [63]. Complex TM treatment was applied in 5 studies [39, 42, 45, 48, 62]. 

The duration of the case studies/series ranged widely from several days to 13.5 years [40]. Padma 28 was investigated in 4 case studies [40, 72–74], Padma Lax in 1 [75], and complex TM in another [76].

All studies included a total of 4684 patients, ranging from 1 to 967 per trial (mean = 117, SD = 187). Ten studies did not state the patients' sex (*n* = 1648, 35.2% of all patients in the present review) [40–42, 47, 56, 63, 65, 67, 71, 77]. From the other studies, 1080 patients (23.1%) were male and 1956 (41.8%) female. Data on age was available in 31 of 39 studies. Children (age 10 months to 16 years, *n* = 955) only were included in 5 studies [46, 64, 65, 70, 71]. Only 2 studies reported on ethnicity (Tibetan patients in both) [42, 58]. In 32 studies, dropouts were reported ranging from 0% (15 studies) to 53% [45] with a mean dropout rate of 15%. In 21 of the 28 trials of Padma 28 or Padma Lax, the mean drop out rate was 6%.

The checklist results for quality assessment are presented at item level in Table 3 for each study. Depending on study type and setting, 10 to 26 items could be answered. Had the assessment been for HTA purposes, only 1 case study [76] and 1 RCT [55] would have been eligible for inclusion in a HTA. Ignoring only one item (G2, provision of confidence intervals) would have raised that number to 13, including 8 RCTs that the Jadad score rated as good or very good quality. The Jadad score of the 15 RCTs (Table 4) reached a mean ± SD of 3.40 ± 1.35 (median = 4). Randomization scored 1.40 ± 0.51 (median = 1), blinding 1.20 ± 1.01 (median = 2), and drop-out reporting 0.80 ± 0.41 (median = 1). Studies on Padma 28 or Padma Lax had higher Jadad scores than studies on other treatments: 3.70 ± 1.06 (median = 4) versus 2.60 ± 1.51 (median = 2). 

All studies followed conventional “Western” medical diagnoses. Additional traditional TM diagnostics were recorded in 11 studies that investigated the traditional multimodal treatment. In 9 of them, the Tibetan diagnosis was used to plan the therapy [39, 42, 43, 45, 48, 58, 59, 62, 76].

Thirty studies including 3497 patients (74.7% from all included studies) investigated single formulations: Padma 28 (*n* = 25 studies), Padma Lax (3), Byu-Dmar 13 (1), and Zhi Byed 11 (1). The complex traditional Tibetan treatment was studied in 9 trials that included a total of 1140 (24.3%) patients. Here, and in the Padma 28 studies, the treated conditions varied widely. For example, Padma 28 was investigated for arteriosclerosis, infections, neurological disorders, venous insufficiency, arthritis, and hypercholesteraemia.

Assessed outcomes included clinical outcomes such as symptom scales (*n* = 37 studies), laboratory tests (19), clinical tests (such as ankle/brachial pressure index, blood pressure, or weight; 9), and other (9), such as microbiology, histology, or the need for conventional medication. The authors drew positive conclusions on their data in 34 studies. In 2 RCTs, TM was found to be inferior to conventional medicine, but better than placebo [44, 46]. In one study, only 1 of 5 outcomes improved [60]; in 2 studies the primary outcome did not change significantly while secondary outcomes did [42, 52]. The comparison of the traditional and a not further specified “special” Tibetan medicine [59] resulted in comparable clinical improvements. The remaining studies found no significant differences to controls [49, 65], or their authors were doubtful about the observed effects [39]. Statements about adverse effects were included in 23 studies, in 11 of them no adverse effects were reported, and 2 studies did not mention the number of patients with adverse effects [39, 53]. The remaining 10 studies reported adverse effects with a range from 5% to 55% of the patients.

Some disease groups were researched in several trials. Peripheral arterial occlusive disease was treated with Padma 28 in 9 studies (6 RCTs, (the first study in [38], [51–55])) 1 observation study, (the second study in [38]) and 2 case studies [40, 72]. Maximum walking distance increased in 5 studies (the first study in [38], and [51, 53–55]). Both case studies and the observational study reported a general clinical improvement. The ankle/brachial pressure index in 1 RCT [52] was unchanged. All authors made a positive conclusion regarding Padma 28.

Five studies (3 non-randomized controlled trials [46, 53, 65] and 2 observation studies [70, 71]) investigated Padma 28 for recurrent respiratory tract infections in children. Improvements were seen for frequency of infections [70, 71] or spontaneous bacterial activity [64]. In 1 of the controlled trials, no significant difference to standard therapy was found [65], and in another study, inferiority to other therapies was reported [46].

Osteoarthritis or rheumatoid arthritis was treated in three trials: 1 RCT [58] and 1 observational study [62] with the traditional multimodal approach, and with Padma 28 in 1 controlled trial [66]. All studies reported pre-/post-improvements or superiority to controls regarding symptom severity. 

Padma Lax in chronic constipation was the subject of three studies (1 RCT [57], 1 controlled trial [75], and 1 observational study [41]). All reported clinical improvements. 

In 3 other trials, hepatitis B patients were either treated with a “special” TM (that was not further specified) in comparison to traditional TM (1 RCT [59]) or with Padma 28 (2 observational studies [47, 69]). All publications reported positive results for laboratory outcomes. The comparison of traditional and “special” traditional TM found comparable improvements but did not achieve seroconversions. 

## 4. Discussion

In this paper, we presented an overview of the clinical research on traditional Tibetan medicine (TM) that is currently available in the West. Three quarters of the included studies tested single formulations, most of them products of a single company. One quarter investigated the traditional multimodal TM approach. Studies were very heterogeneous regarding study type and size, treated conditions, treatments, measured outcomes, and quality.

In this, to our knowledge, first systematic overview of clinical TM research available in the West, we tried to minimize subjectivity using pre-defined systematic methods wherever possible (data extraction sheets, established quality assessment tools). However, the small number of trials scattered over a whole medical system and very heterogeneous treated diseases prohibited more formal or in-depth analyses.

Despite the broad literature search, some studies may not have been identified, for various reasons. Although Mongolian and Tibetan medicine are not completely identical, we have included “mongolian” in the search terms in order to find as much relevant literature as possible. We did not search for single TM interventions such as bathing or bloodletting and assumed that they are well covered under the umbrella term “medicine.” Although we detected with this search a study on Tibetan yoga [60], we possibly missed other studies. Furthermore, publication bias could have had occurred, as some papers [11, 15, 58] indicated the existence of studies that have not been published (or at least not in indexed journals) [77–82]. Several papers were not identified by our search strategy in the literature databases, but could have been found searching for “Padma 28” or “Padma Lax.” Clearer labelling of TM studies in the future would be helpful. On the other hand, our search seems to have been partly redundant, as all identified publications could have been found with fewer search terms. The main limitation is that our language restriction excluded articles in Russian, Tibetan, and Chinese. This literature was not accessible for us. Furthermore, we learned from our field work and from discussions with Western and Chinese manufacturers during an interdisciplinary symposium on TM [16] that most literature on clinical research published in Tibetan is not available in indexed journals and that most research published in Chinese addresses preclinical questions.

The evaluated literature presented a high number of studies without a control group. Only a few single products were subject to in-depth investigation. Both facts indicate an early stage of research in a new and largely unexplored field where only few focused inquiries exist. The predominating countries of origin (>2/3 European) and the 70% of studies on Padma products among the included literature are consequences of the language restrictions of our search as well as of the historical development of TM utilization in the West. Although they are prescribed in a standardized and nonindividualized fashion, the Padma products are a genuine Tibetan medication according to manufacturers, study authors, and independent experts [17, 83, 84]. Adaptation of constituents to local situation and ecology is an accepted practice in TM. It was done in one study when Tibetan physicians reduced the traditional Byu-Dmar 25 by 12 ingredients to comply with Tibetan pharmacopoeia and European regulations, resulting in Byu-Dmar 13 [63]. A similar strategy might have been used in two other studies [39, 62]. 

The heterogeneous nature of the included studies demanded the use of quality assessment instruments that were suitable for diverse study designs, but have the general disadvantage of allowing only rough estimates of the assessed quality. Nevertheless, they allowed spotting the more obvious deficiencies that are symptomatic of research at an early stage and that future research can avoid with improved methodology on the grounds of evidence-based medicine. Case studies and observational studies are useful to gather information on traditional usage and settings and to identify areas where controlled studies seem promising. Then, to provide higher-level evidence, more RCTs will be needed. Methodological issues such as small samples, insufficiently described populations in many studies, pre-/post-comparisons of treatment within a group, or comparator treatments without clinical relevance all indicate that TM research as seen through the Western literature is still at a nascent stage. Furthermore, the quality of most studies and the heterogeneity of interventions and outcomes make clear conclusions impossible. 

## 5. Conclusion

The clinical research on traditional Tibetan medicine (TM) that is available in Western industrialized countries is scarce and scattered over a whole medical system, but shows interesting results. Better research methodology should be applied, and larger trials are needed, as is a general overview of traditional usage to inform future clinical research.

## Figures and Tables

**Table 1:** DIMDI HTA checklist items.

Item*	Item no. (label)**
(A) Selection of participants	Participants
(1) Were the criteria for in-/exclusion defined sufficiently and clearly?	A1 (in-/exclusion)
(2) Were the criteria for in-/exclusion defined before intervention?	A2 (predefined)
(3) Was the health status recorded in a valid and reliable way?	A3 (health status)
(4) Were the diagnostic criteria of the disease described?	A4 (diagnostic criteria)
(5) Were the studied/exposed patients representative for the majority of the exposed population or the “standard users” of the intervention?	A5 (representativity)

(B) Allocation and study participation	Allocation
(1) Were the exposed/cases and nonexposed/controls from the same base population?	B1 (basic population)
(2) Were intervention/exposed and control/nonexposed groups comparable at baseline?	B2 (comparable)
(3) Was allocation randomized, with a standardized procedure?	B3 (randomization)
(4) Was randomization blinded?	B4 (blinded randomization)
(5) Were known/possible confounders considered at baseline?	B5 (confounders)

(C) Intervention/exposition	Intervention
(1) Were intervention or exposition recorded in a valid, reliable, and similar way?	C1 (recording)
(2) Apart from intervention, were intervention and control groups treated similarly?	C2 (similar treatment)
(3) In case of other treatments, were they recorded in a valid and reliable way?	C3 (other treatments)
(4) For RCTs: were placebos used for the control group?	C4 (placebo use)
(5) For RCTs: was the way of placebo administration documented?	C5 (placebo documented)

(D) Study administration	Administration
(1) Are there indications for “overmatching”?	D1 (overmatching)
(2) In multicentre studies: were the diagnostic and therapeutic methods and the outcome recording in the centres identical?	D2 (multicentre)
(3) Was if assured that participants did not crossover between intervention and control group?	D3 (no crossover)

(E) Outcome recording	Outcome
(1) Were patient-centred outcome parameters used?	E1 (patient-centred)
(2) Were the outcomes recorded in a valid and reliable way?	E2 (recording)
(3) Was outcome recording blinded?	E3 (blinded outcomes)
(4) For case series: was the distribution of prognostic factors recorded sufficiently?	E4 (prognostic factors)

(F) Drop-outs	Drop-outs
(1) Was the response rate in intervention/control group sufficient, or, for cohort studies, could a sufficient part of the cohort be tracked for the full study duration?	F1 (evaluable number)
(2) Were the reasons for the dropouts of participants stated?	F2 (reasons)
(3) Were the outcomes of dropouts described and included in the analysis?	F3 (outcomes)
(4) If differences were found: were they significant?	F4 (significance)
(5) If differences were found: were they relevant?	F5 (relevance)

(G) Statistical analysis	Statistics
(1) Were the described analytic methods correct and the information sufficient for a flawless analysis?	G1 (correct)
(2) Were confidence intervals given for means and for significance tests?	G2 (CIs given)
(3) Were the results presented in graphical form, and were the underlying values stated?	G3 (graphics)

*Translated from [35], **used in Table 3.

**Table 2:** Included studies.

Study Type* Country	Disease (diagnostic system)**	Participants (mean age), drop-outs***	Duration of intervention or study kind, dose of intervention****	(1) Main outcome (2) Other outcomes	Notes
Aschoff et al. 1997 OS Germany	Migraine (BM)	I: 22; D: 0	6 months (and longer?) Byu-Dmar 13 jewel pill, 1 U/d	(1) Severity of attacks reduced by 82% (2) Frequency of attacks unchanged; less use of analgesics in most participants	Very brief documentation; only subjective outcomes

Bommeli et al. 2001 rCS (MC) Switzerland	Various (78% patients w/arteriosclerosis) (BM, TM)	I: 147; D: 18	From few days to 13.5 years P28, varying doses (~50% of patients 3 × 2 U/d)	(1) Improvement of complaints in % of patients: peripheral artery occlusive disease in 94%, coronary heart disease in 92%, chronic venous insufficiency in 91%, arthrosis in 80%	Patients from 15 physicians, no demographics, no monotherapy, success not clearly attributable to P28

Brunner-La Roccaet al. 2005 RCT (5) Switzerland	Mild hypercholesterolaemia (BM)	I: 30; C: 30; D: 0	4 weeks + 15 d followup I: P28, 3 × 2 U/d C: potato starch	(1) Total cholesterol unchanged (2) Other blood lipids unchanged	Participants not typical patients

Brzosko et al. 1991 CT (4 arms) Poland	Chronic juvenile arthritis (BM)	I1: 12 (11 years); I2: 7; C1: 10 (healthy); C2: 10 (in remission)	I1: 6 weeks; I2: 4 weeks I1: P28, 2–4 U/d I2: Thymus extract, 1 suppositorium/day	(1) Joint pain and swelling (Ritchie Index): improved in 75%–83% of P28 patients, in 86% of thymus extract patients (2) Improvement (compared to healthy control) of sedimentation rate, IgG, IgM, seromucoid, CD8-Lymphocytes, CD4/CD8-quotient	Control is no standard therapy; comparison with healthy probands; immunological parameters not very relevant for contemporary diagnostics

Brzosko and Jankowski 1992 OS (MC) Poland	Hepatitis B (BM)	I: 178 including 52 children	2 years (intervention), 10 years (study) I: P28, 3 × 2 U/d	(1) “Biochemical markers” (not specified) improved in ~90% (2) Improvements in T lymphocytes (CD3, CD4, CD8, and CD4/CD8) in 90%, hepatocellular virus eliminated in 15%, improvements in immunohistochemistry (HBe-Ak increase in 70%), and clinical findings (in 90%)	Very brief description of patients and outcomes; no statement about other therapies

Changbar 1998 CS India	Chronic aplastic anaemia (BM, TM)	I: 1 man (63); D: 0	15 months Rinchen yusnying 25 special, 1 on alternating days; Zhiru, 2× on alternating days; Gur gum 8 special, 4 × /d; Se ‘bru kun bde, 3× /d; A gar 8, 4 × /d; dietary recommendations	(1) Haemoglobin (increase from 3.1 to 10.4 mg/dL) (2) Clinical improvement, reduction of comedication	

Cohen et al. 2004 RCT (2) USA	Mental symptoms accompanying lymphomas (BM)	I: 19; C: 19; D: 9	7 weeks + 3 months follow-up 7 weekly sessions of guided yoga (Tsa lung trul khor yoga)	(1) Sleep disorder improved (2) Despair, anxiety, depression, fatigue not significant, patient's appraisal positive	Many outcomes in small population increased probability of significant results caused by random variations; high drop-out rate; low compliance

Feldhaus 2004 CS Switzerland	Peripheral arterial occlusive disease (BM, unspecified CAM)	I: 1 woman (61); D: 0	1 year P28, 3 × 2 U/d; intestinal cleansing (intestinal hydrotherapy and microbacterial treatment), chelation therapy, oxygenation therapy, orthomolecular treatment, IV treatment with ribonucleic acid	(1) General condition much improved after 8 months (2) Walking distance improved (<100 m to >2000 m)	No attribution of effect to TM possible

Feldhaus 2006 CS Switzerland	Chronic constipation of tetraplegic patients (BM, unspecified CAM)	I: 3; D: 0	1–3 months I: PL, 1 × 1-2 U/d; intestinal cleansing (intestinal hydrotherapy and micro bacterial treatment), chelation therapy, other CAM	(1) Constipation cured in all cases	No attribution of effect to TM possible

Flück and Bubb 1970 OS (MC) Switzerland	Chronic constipation (BM)	I: 285 (256 outpatients, 29 inpatients)	“Several” weeks PL, 1 × 1 U/d	(1) Symptoms improved in 82% (2) Unwanted effects in 6.3%	Insufficient description of population, inclusion criteria, and diagnostics

Füllemann 2006 OS Switzerland	Chronic dental pulpitis (BM)	I: 53; D: 4	15 days P28, 2 × 2 U/d	(1) Pain-free within 1 month in 55% (2) Extraction or root canal treatment not necessary in 82%	Comparison with expectation from experience; 4 drop-outs because of incompliance might have caused false positive result

Gladysz et al. 1993 OS Poland	Hepatitis B (BM)	I: 34	12 months P28, 3 × 2 U/d	(1) Serological and liver function parameters improved in 76.5%, liver biopsy improved in 55.9% (2) Other parameters (GGT, GPT, bilirubin, and albumin) unchanged	Authors claim elimination potential for HBeAg and HBV-DNA similar to interferon standard therapy; unwanted effects not stated

Günsche 2005 CS Switzerland	Bipolar Disorder (BM)	I: 1 woman (44); D: 0	11 months P28, 3 × 2 U/d for 6 weeks, then 3 × 1/d	(1) and (2) Daytime sleepiness, concentration difficulties, and apathy much improved within 6 weeks, cured after 11 months	Only subjective outcomes

Hürlimann 1979/1 RCT (3) Switzerland	Peripheral arterial occlusive disease (BM)	I: 13; C: 11; D: 0	12 weeks I: P28, 3 × 2 U/d C: Placebo	(1) Pain free walking distance improved by 54% (2) Other symptoms improved in 69%, no change in plethysmography	Good study design, homogenous groups, very brief presentation of results, valid results

Hürlimann 1979/2 OS Switzerland	Peripheral arterial occlusive disease (BM)	I: 10; D: 0	Duration not stated P28, 3 × 2 U/d.	(1) Rest pain improved in 70%	Very brief presentation, duration not stated

Jankowski et al. 1986 OS Poland	Recurrent respiratory tract infections (BM)	I: 61 (2 years); D: 0	8 weeks P28, 3 × 1 U/d or 3 × 0.5 U/d depending on age, 4 weeks P28—2 weeks pause—2 weeks P28	(1) Frequency and intensity of infections reduced in 80% (2) Immunoglobulins and B cells unchanged, T cells normalized, phagocytic activity of leucocytes increased, appetite increased	Immunological analysis did not include all participants

Jankowski et al. 1991 CT Poland	Recurrent respiratory tract infections (BM)	I: 19; C: 10 (healthy); (3 years); D: 0	8 weeks P28, 3 × 1 U/d, 4 weeks P28—2 weeks pause—2 weeks P28	(1) Bactericide index (“spontaneous bactericidal activity”) improved in 84%	Effect not clearly attributable because of healthy controls; tested bacteria not typical for disease; unusual outcome parameter

Jankowski et al. 1992 OS Poland	Recurrent respiratory tract infections (BM)	I: 305 (4 years)	10 weeks P28, 3 × 1 U P28 or 3 × 0.5 U depending on age	(1) Frequency and intensity of infections reduced in 72% (2) Increase in CD2+, CD4+ lymphocytes, and CD4/CD8 quotient	Possibly republished data from earlier studies; immunological results from 48 participants only (randomized?)

Korwin-Piotrowskaet al. 1992 RCT (2) Poland	Multiple Sclerosis (BM)	I: 50; C: 50; D: 0	12 months I: P28, 3 × 2 U/d C: Placebo, symptomatic treatment	(1) Clinical course (relapse frequency or progression) improved in 44% (2) Evoked potentials: visual improved in 33%, acoustic unchanged	Other treatment in placebo group

Leeman et al. 2001 OS USA	Breast cancer (BM, TM)	I: 11; DI: 2	1 year 2–4 herbal preparations, 2–6×/d; diet, lifestyle regulation, prayer; every 3–4 months adjustment of prescription	(1) No unwanted effects grade III or IV (2) 1 patient's tumor regressed, 2 were stable for >12 months, 6 progressed	No peer-reviewed publication; no statements about drop-out's outcomes (possibly disease progress)

Li 2001 OS (MC) Lhasa Prefecture, China	Helicobacter pylori associated gastritis (BM, TM)	I: 86	Max. 8 weeks, follow-up of 24 patients after 5 months TM, max. 8 weeks	(1) Helicobacter test not changed (2) Clinical parameters improved in 76.3%–100% (depending on category), symptom intensity improved	Therapy according to Tibetan diagnostics in 9 “medication groups”; selection of followup group not stated

Mansfeld 1988 CT Switzerland	Recurrent respiratory tract infections (BM)	I: 218; C: 205; (11 years); D: 3	6 weeks, then observation for 6–12 months I: P28, 3 × 1 U/d, biomedicine when needed, mountain air cure C: biomedicine when needed, mountain air cure	(1) Frequency and severity of infections tended to improve (not significant) (2) Immunoglobulines and inflammation parameters not significant	Parents assessed infection severity; other therapies might have masked P28 effect

Mehlsen et al. 1995 RCT (5) Denmark	Peripheral arterial occlusive disease	I: 20; C: 20; D: 4	4 months I: P28, 2 × 2 U/d C: gelatine	(1) Max. walking distance improved (2) Pain-free walking distance improved, no change in blood pressure and blood pressure ratio ankle/upper arm	Excellent study design

Miller et al. 2009 RCT (5) (MC) Lhasa Prefecture, China	Post-partum haemorrhage (BM, TM)	I: 480; C: 487; D: 7	Single dose I: Zhi Byed 11, 3 U, and placebo C: Misoprostol, 600 *μ*g, and placebo	(1) Misoprostol superior to Zhi Byed 11 for: Hemorrhage, maternal death, need for uterotonics (2) No significant difference for mean and median blood loss	

Namdul et al. 2001 RCT (1) (MC) India	Type 2 Diabetes (BM, TM)	I: 100; C: 100; D: 88 (64 after 12 weeks)	24 weeks I: Kyura-6, Aru-18, Yung-4, and Sugmel-19, daily + life style regulation + diet according to American Diabetes Association C: life style regulation + diet as above	(1) Fasting blood glucose reduced (2) Postprandial blood glucose and HbA1c reduced, weight, blood pressure, and blood lipids unchanged	Intervention group more ill despite randomization; values of intervention group taken as baseline; high drop-out rate without further analyses

Neshar 2000 OS India	Diabetes mellitus (BM, TM)	I: 82; D: 0 (study of patient files)	Min. 6 months Yung-4, Kyuru-6, Chinni-Aru-18, and Sugmel-10, daily + lifestyle and diet regulation	(1) Blood glucose improved in 70%, stabilized in 100% (2) Improvements in subjective symptoms (92%), and need for biomedicine in 68%	Regarding general improvement discrimination between TM alone or with additional biomedicine: it is not clear whether biomedicine was given at baseline or became necessary during study; most data refer to a subpopulation of 24 that is not described: selection bias?

Neshar 2007 OS India (MC)	Cancer (BM, TM)	I: 647; D: 340	Varying duration Traditional TM (not further specified)	(1) General health state much improved (2) Improvements in progression, infections, pain, side effects of chemotherapy and radiation therapy	Selection of patients not representative, high drop-out rate

Pauwvliet et al. 1997 OS Netherlands	Rheumatic disorders (BM, TM)	I: 35; D: 7	6 months Traditional TM (not further specified)	(1) Severity of disease improved (2) Improvements in pain, number of diseased parts general well-being, and mental complaints	High drop-out rate, 4 of them because of aggravation; prepublication without laboratory data

Prusek et al. 1987 CT (6 arms) (MC) Poland	Recurrent respiratory tract infections (BM)	I: 30; C1: 23; C2: 10; C3: 29; C4: 25; C5: 20; (4 years); D: 0	11 months I: P28, 3 × 1 U/d for 1 month C1: levamisole, 3 mg/kg: for 2 × 3 d C2: thymus factorx, 1 mg/kg for 3 weeks C3: bacterial lysate, 3.5 mg/d for 3 × 10 d C4: climate cure for 6 weeks C5: healthy probands	(1) Frequency and severity of infections improved in 57% (less than controls) (2) Immunoglobulines not changed, T cells improved	Comparability of groups unclear (allocation by clinical indication); statistical evaluation not sufficient

Rüttgers 2004 CS Switzerland	Chronic venous insufficiency (BM)	I: 1; D: 0	3 months and follow-up P28, 3 × 1 U/d and biomedical standard (no primarily angiological) therapy	(1) Inflammation improved (2) Oedema and pain improved; remission for >6 months; healing faster under P28	

Ryan 1997 RCT (3) India	Arthritis (BM, TM)	I: 15; C: 15; D: 2	3 months I: traditional TM (not further specified) C: biomedical treatment	(1) Motility of extremities improved, in 86% of the matched pairs the TM patient better than respective control	Inclusion by Tibetan diagnosis; no further details to matched pairs; only two pairs of arthritis patients

Sallon et al. 1998 RCT (4) Israel	Peripheral arterial occlusive disease (BM)	I: 37; C: 35; D: 13	6 months I: P28, 2 × 2 U/d C: potato starch	(1) Ankle-brachial-index unchanged (2) Improved: pressure decrease, ischaemia time, and patient's assessment	

Sallon et al. 2002 RCT (4) Israel	Chronic constipation (BM)	I: 42; C: 38; D: 19	12 weeks I: PL, 2 × 2 U/d, C: potato starch	(1) Improved intestinal passage (2) Improved abdominal pain (physician's assessment) and everyday activity (patient's assessment)	Comprehensive study documentation

Samochowiec et al. 1987 RCT (4) Poland	Peripheral arterial occlusive disease (BM)	I: 55; C: 45	4 months I: P28, 2 × 2 U/d C: lactose	(1) Improved max. walking distance (2) Upper arm blood pressure unchanged, improved: total blood lipids, *β*-lipoproteins, thrombocyte aggregation threshold	No patient demographics; comparison only to baseline, not between groups

Sangmo et al. 2007 RCT (2) India	Hepatitis B (BM, TM)	I: 24; C: 25; D: 1	6 months I: Special TM, (not further described) C: Traditional TM	(1) No differences between groups (2) Both groups tended to improvements in liver function and improved clinically	Special TM group more ill at baseline; almost no appraisal of results; possibly overtesting; very comprehensive documentation also of Tibetan diagnostics

Schleicher 1990 OS Germany	Acquired immune deficiency syndrome (BM)	I: 15; D: 5	6 months P28, 3 × 3 U/d	(1) Total T cells stabilized (2) Stabilized: suppressor-cytotoxic cells, helper-inducer cells, and lymphocytes; unchanged: B cells and killer cells; increase in granulocytes and phagocytosis	No patient-centred parameters; prognostically most relevant CD4 cell count and viral load not documented

Schrader et al. 1985 RCT (4) Switzerland	Peripheral arterial occlusive disease (BM)	I: 27; C: 26; D: 10	4 months I: P28, 3 × 2 U/d C: lactose	(1) Improved max. walking distance (2) Improved pain-free walking distance	

Smulski and Wojcicki 1994 RCT (5) Poland	Peripheral arterial occlusive disease (BM, TM)	I: 50; C: 50; D: 7	4 months I: P28, 2 × 2 U/d C: lactose	(1) Max. walking distance improved (2) Patient's assessment more positive, improved total blood lipids, triglycerides, low density lipoproteins	Comparison of groups only for walking distance

Split et al. 1998 RCT (2) Poland	Apoplexy (BM)	I: 60; C: 60	14 days I: P28, 3 × 2 U/d + biomedical standard therapy C: biomedical standard therapy	(1) Better general status (Karnofsky functional efficiency scale, KFES) (2) Better T cells, B cells, and clinical progress	Age not stated, no blinding, no placebo, comparison only understandable for KFES, therapy effect not discernible from placebo effect

Wojcicki et al. 1986 CT Poland	Coronary heart disease, angina pectoris (BM)	I: 50	6 weeks Placebo, 2 weeks—P28, 2 × 2 U/d, 2 weeks—placebo, 2 weeks	(1) Nitroglycerine need reduced (2) Improvement of exercise capacity, platelet aggregation, and blood lipids	No randomization (contrary to publication statement); description difficult to understand; selection of patients from larger population not clear; short verum period

*(r)CS: (retrospective) case study; CT: controlled trial (not randomized); OS: observational study; RCT: randomized controlled trial (with Jadad sum score); MC: multicentre study.

**BM: Biomedicine (the “Western” “conventional” medicine); TM: Tibetan medicine; CAM: complementary or alternative medicine.

***I: intervention group (TM); C: control group (other treatment, placebo); D: total dropouts.

****U: unit (tablet, capsule, or pill); /d: per day; P28: Padma 28; PL: Padma Lax.

**Table 3:** DIMDI HTA checklist results.

				Participants					Allocation					Intervention					Administration			Outcome				Drop-outs					Statistics		
	Study		Item no. (label) *	A1 (in-/exclusion)	A2 (pre-defined)	A3 (health status)	A4 (diagnostic criteria)	A5 (representativity)	B1 (basic population)	B2 (comparable)	B3 (randomization)	B4 (blinded randomization)	B5 (confounders)	C1 (recording)	C2 (similar treatment)	C3 (other treatments)	C4 (placebo use)	C5 (placebo documented)	D1 (overmatching)	D2 (multicentre)	D3 (no crossover)	E1 (patient-centred)	E2 (recording)	E3 (blinded outcomes)	E4 (prognostic factors)	F1 (evaluable number)	F2 (reasons)	F3 (outcomes)	F4 (significance)	F5 (relevance)	G1 (correct)	G2 (CIs given)	G3 (graphics)
	Brunner-LaRocca et al. 2005			Y	Y	Y	Y	Y	Y	Y	Y	Y	Y	Y	Y	·	Y	Y	N	·	Y	N	Y	Y	·	Y	Y	Y	·	·	Y	N	Y
	Cohen et al. 2004			Y	Y	Y	N	Y	Y	Y	?	Y	Y	Y	Y	·	N	·	N	·	Y	Y	Y	N	·	?	Y	N	·	·	Y	Y	N
	Hürlimann1979/1			Y	Y	Y	Y	Y	Y	Y	Y	Y	Y	Y	Y	·	Y	Y	N	·	Y	Y	Y	?	·	Y	·	·	·	·	Y	N	N
	Korwin-Piotrowska et al. 1992			Y	Y	Y	N	Y	Y	Y	?	N	Y	Y	N	N	N	·	N	·	Y	Y	Y	?	·	Y	·	·	·	·	Y	N	N
	Mehlsen et al. 1995			Y	Y	Y	Y	Y	Y	Y	Y	Y	Y	Y	Y	·	Y	Y	N	·	Y	Y	Y	Y	·	Y	Y	N	·	·	Y	Y	N
	Miller2009			Y	Y	Y	Y	Y	Y	Y	?	Y	Y	Y	Y	·	Y	Y	N	?	Y	Y	Y	Y	·	Y	Y	N	·	·	Y	N	N
Randomized controlled trials	Namdul et al. 2001			Y	Y	Y	Y	Y	Y	Y	?	?	?	Y	Y	·	N	·	N	Y	Y	Y	Y	?	·	Y	N	N	·	·	Y	N	N
	Ryan1997			Y	Y	Y	Y	?	Y	Y	?	?	Y	Y	Y	Y	N	·	N	·	Y	Y	Y	N	·	Y	N	N	·	·	Y	N	N
	Sallon et al. 1998			Y	Y	Y	Y	Y	Y	Y	Y	Y	Y	Y	Y	·	Y	Y	N	·	Y	Y	Y	Y	·	?	Y	N	·	·	Y	Y	Y
	Sallon et al. 2002			Y	Y	Y	Y	Y	Y	Y	Y	Y	Y	Y	Y	·	Y	Y	N	·	Y	Y	Y	Y	·	Y	Y	N	·	·	Y	N	Y
	Samochowiec et al. 1987			Y	Y	Y	Y	Y	Y	Y	Y	Y	Y	Y	Y	·	Y	Y	N	·	Y	Y	Y	Y	·	Y	·	·	·	·	Y	N	Y
	Sangmo et al. 2007			Y	Y	Y	Y	Y	Y	Y	?	?	?	N	Y	·	N	·	N	·	Y	Y	Y	N	·	Y	Y	N	·	·	Y	N	N
	Schrader et al. 1985			Y	Y	Y	Y	Y	Y	Y	Y	Y	Y	Y	Y	·	Y	Y	N	·	Y	Y	Y	Y	·	Y	Y	N	·	·	Y	N	N
	Smulski and Wojcicki 1994			Y	Y	Y	Y	Y	Y	Y	Y	Y	Y	Y	Y	·	Y	Y	N	·	Y	Y	Y	Y	·	Y	Y	N	·	·	Y	Y	Y
	Split et al. 1998			Y	Y	N	N	?	Y	Y	?	?	?	Y	Y	·	N	·	N	·	Y	Y	Y	?	·	Y	·	·	·	·	Y	N	N

	Brzosko et al. 1991			Y	Y	Y	Y	Y	Y	N	N	·	N	Y	Y	·	·	·	N	·	Y	Y	Y	N	·	Y	·	·	·	·	Y	N	N
Controlled trials	Jankowski et al. 1991			Y	Y	Y	Y	Y	N	N	N	·	N	Y	N	·	·	·	N	·	Y	N	Y	N	·	Y	·	·	·	·	Y	N	N
	Mansfeld1988			Y	Y	Y	N	Y	Y	?	N	·	?	Y	Y	·	·	·	N	·	Y	Y	?	N	·	Y	·	·	·	·	Y	N	Y
	Prusek et al. 1987			Y	Y	Y	Y	Y	Y	N	N	·	N	Y	?	·	·	·	N	N	Y	Y	?	N	·	Y	·	·	·	·	Y	N	N
	Wojcicki et al. 1986			Y	Y	Y	Y	Y	Y	Y	N	·	Y	Y	Y	·	·	·	N	·	Y	Y	Y	N	·	Y	·	·	·	·	Y	N	N

	Aschoff et al. 1997			Y	Y	Y	Y	Y	·	·	·	·	Y	Y	N	N	·	·	·	·	·	Y	Y	·	·	Y	·	·	·	·	Y	N	N
	Brzosko and Jankowski 1992			Y	Y	?	N	Y	·	·	·	·	?	Y	?	·	·	·	·	·	·	Y	Y	·	·	Y	·	·	·	·	Y	N	N
	Flück and Bubb 1970			Y	Y	Y	Y	Y	·	·	·	·	N	Y	?	·	·	·	·	Y	·	Y	Y	·	·	Y	·	·	·	·	Y	N	N
	Füllemann 2006			Y	Y	Y	Y	Y	·	·	·	·	Y	Y	N	N	·	·	·	·	·	Y	Y	·	·	Y	Y	Y	N	N	Y	N	N
	Gladysz et al. 1993			Y	Y	Y	Y	Y	·	·	·	·	Y	Y	Y	·	·	·	·	·	·	N	Y	·	·	Y	·	·	·	·	Y	N	N
	Hürlimann 1979/2			Y	Y	Y	Y	Y	·	·	·	·	?	Y	?	·	·	·	·	·	·	Y	N	·	·	Y	·	·	·	·	Y	N	N
Observation studies	Jankowski et al. 1986			Y	Y	Y	Y	Y	·	·	·	·	N	Y	?	·	·	·	·	·	·	Y	Y	·	·	Y	·	·	·	·	Y	Y	N
	Jankowski et al. 1992			Y	Y	Y	Y	Y	·	·	·	·	N	Y	Y	·	·	·	·	·	·	Y	Y	·	·	Y	·	·	·	·	Y	N	N
	Leeman at al. 2001			Y	Y	Y	N	Y	·	·	·	·	N	N	N	N	·	·	·	·	·	Y	Y	·	·	Y	N	N	·	·	Y	N	N
	Li 2001			Y	Y	Y	Y	Y	·	·	·	·	Y	N	Y	·	·	·	·	Y	·	Y	Y	·	·	Y	·	·	·	·	Y	N	N
	Neshar 2000			Y	N	Y	Y	Y	·	·	·	·	N	Y	·	·	·	·	·	·	·	Y	N	·	·	?	·	·	·	·	N	N	N
	Neshar 2007			Y	Y	?	Y	?	·	·	·	·	?	Y	·	·	·	·	·	Y	·	Y	Y	·	N	Y	N	N	·	·	Y	N	N
	Pauwvlietet al. 1997			Y	Y	Y	Y	Y	·	·	·	·	?	Y	N	Y	·	·	·	·	·	Y	Y	·	·	Y	·	·	·	·	Y	N	N
	Schleicher 1990			Y	Y	Y	Y	Y	·	·	·	·	N	Y	?	N	·	·	·	·	·	N	Y	·	·	?	·	·	·	·	Y	N	N

	Bommeli et al. 2001			Y	Y	Y	Y	Y	·	·	·	·	Y	Y	N	N	·	·	·	Y	·	Y	Y	·	N	Y	Y	N	·	·	Y	N	N
	Changbar 1998			Y	·	Y	Y	N	·	·	·	·	·	Y	·	·	·	Y	·	·	·	Y	Y	·	N	Y	·	·	·	·	·	·	N
Case studies	Feldhaus 2004			Y	·	Y	Y	N	·	·	·	·	?	Y	·	·	·	·	·	·	·	Y	Y	·	N	Y	·	·	·	·	·	·	N
	Feldhaus 2006			Y	Y	Y	Y	Y	·	·	·	·	?	Y	N	·	·	·	·	·	·	Y	Y	·	N	Y	·	·	·	·	·	·	N
	Günsche 2005			Y	·	Y	Y	N	·	·	·	·	Y	Y	·	·	·	·	·	·	·	Y	N	·	N	Y	·	·	·	·	·	·	N
	Rüttgers 2004			Y	·	Y	Y	N	·	·	·	·	N	Y	·	·	·	·	·	·	·	Y	N	·	N	Y	·	·	·	·	·	·	N

Y: yes; N: no; ?: unclear/not stated; ·: not applicable.

*Full item text in Table 1.

**Table 4:** Jadad Score Results for Included RCTs.

	Randomization	Blinding	Drop-outs	Sum score
Brunner-La Rocca et al. 2005	2	2	1	5
Cohen et al. 2004	2	0	0	2
Hürlimann 1979/1	1	2	0	3
Korwin-Piotrowska et al. 1992	1	0	1	2
Mehlsen et al. 1995	2	2	1	5
Miller 2009	2	2	1	5
Namdul et al. 2001	1	0	0	1
Ryan 1997	2	0	1	3
Sallon et al. 1998	1	2	1	4
Sallon et al. 2002	1	2	1	4
Samochowiec 1987	1	2	1	4
Sangmo et al. 2007	1	0	1	2
Schrader et al. 1985	1	2	1	4
Smulski and Wojcicki 1994	2	2	1	5
Split et al. 1998	1	0	1	2
